# Multigene phylogenetics of ***Polycephalomyces*** (Ophiocordycipitaceae, Hypocreales), with two new species from Thailand

**DOI:** 10.1038/s41598-018-36792-4

**Published:** 2018-12-27

**Authors:** Yuan-Pin Xiao, Ting-Chi Wen, Sinang Hongsanan, Rajesh Jeewon, J. Jennifer Luangsa-ard, Siraprapa Brooks, Dhanushka Nadeeshan Wanasinghe, Feng-Yao Long, Kevin D. Hyde

**Affiliations:** 1 Engineering Research Center of Southwest Bio-Pharmaceutical Resources, Ministry of Education, Guizhou University, Guiyang, Guizhou Province, 550025 China; 2 Center of Excellence in Fungal Research, Mae Fah Luang University, Chiang Rai, 57100 Thailand; 3 School of Science, Mae Fah Luang University, Chiang Rai, 57100 Thailand; 4 Microbe Interaction and Ecology Laboratory, BIOTEC, NSTDA, 113 Thailand Science Park, Pathum Thani, 12120 Thailand; 5 Department of Health Sciences, Faculty of Science, University of Mauritius, Reduit, 80837 Mauritius

## Abstract

*Polycephalomyces* (Ophiocordycipitaceae) species are found in subtropical regions and are parasitic or hyperparasitic on insects. Two new species, *P. aurantiacus* and *P. marginaliradians*, parasitic on *Ophiocordyceps barnesii* and larva of Cossidae respectively, are introduced in this paper. Morphological comparison with extant species and DNA based phylogenies from analyses of a multigene dataset support the establishment of the new taxa. *Polycephalomyces aurantiacus*, exhibiting a hyperparasitic lifestyle on *Ophiocordyceps barnesii*, differs from other species in producing orange conidia in mass and have longer β-phialides in culture. *Polycephalomyces marginaliradians* differs from other *Ophiocordyceps* species by producing single stromata with a stipe, smaller perithecia and branched α-phialides and catenate α-conidia and is parasitic on Cossidae. A combined nrSSU, nrLSU, ITS, tef-1a, rpb1 and rpb2 sequence data was analysed phylogenetically including *Ophiocordyceps* and *Polycephalomyces* taxa. The new species described herein are clearly distinct from other species in *Polycephalomyces*. We provide a key to the species of *Polycephalomyces* and discuss relevant interspecies relationships.

## Introduction

The genus *Polycephalomyces* was introduced by Kobayasi^1^ to accommodate *P. formosus* Kobayasi (1941), based on its asexual characteristics^2^ and it is presently accommodated in Ophiocordycipitaceae^3^. Phylogenetic placement of *Polycephalomyces* has always been a debate within the clavicipitoid fungi as the taxonomic hypotheses based on host substrate and sexual morph affinities were controversial^1,4,5^. Kepler *et al*.^5^ amended the taxonomic circumscription of *Polycephalomyces* and accepted twelve species (*i.e. P. cuboideus*, *P. cylindrosporus*, *P. ditmarii*, *P. formosus*, *P. kanzashiznus*, *P. nipponicus*, *P. paracubiodeus*, *P. prolificus*, *P. ramosopulvinatus*, *P. ramosus*, *P. ryogamiensis* and *P. tomentosus*) in Ophiocordycipitaceae based on phylogenetic analyses. Later, *P. sinensis*^6^, *P. lianzhouensis*^7^, *P. yunnanensis*^8^, *P. agaricus*^9^ and *P. onorei*^10^ were introduced as new species within *Polycephalomyces* based on morphology and DNA sequence data. Then, Liang *et al*.^11^ introduced a new species *P. ponerae* in the genus *Polycephalomyces*. Based on recent morphological studies and DNA based phylogenetic analyses, *Polycephalomyces* taxa have been segregated in two sister clades within Ophiocordycipitaceae^5,12^. Matočec *et al*.^12^ considered those two clades as two different genera, viz. *Perennicordyceps* Matočec & I. Kušan and *Polycephalomyces*. *Perennicordyceps* comprises four species (i.e. *Pe. cuboidea*, *Pe. paracuboidea*, *Pe. prolifica*, and *Pe. ryogamiensis*), which are characterized by superficial perithecia, and hirsutella-like or acremonium-like asexual morphs^12,13^. *Polycephalomyces* comprised eight species^12^. Maharachchikumbura *et al*.^14,15^ and Wijayawardene *et al*.^3^ maintained *Polycephalomyces* within the Ophiocordycipitaceae.

The sexual morph of *Polycephalomyces* has been recorded as fertile, capitulate, globose, tuberiform to pulvinate stromata and immersed, elongated pyriform perithecia^12^, while the asexual morph has branched or unbranched synnemata, ending up with clavate to spherically flared, hymeniform aggregations of conidiophores, which produce large masses of conidia united in collective globular mucus^12^. There is only one species (*P. lianzhouensis*) reported with both sexual and asexual morphs. *Ophiocordyceps* fungi are ecologically important host species for *Polycephalomyces*, and to date five *Ophiocordyceps* species have been reported to be associated with *Polycephalomyces*^6,8,9,16–18^. At the Engineering Research Center of Southwest Bio-Pharmaceutical Resources (Guizhou University, China) in collaboration with the Center of Excellence in Fungal Research (Mae Fah Luang University, Thailand), we are investigating diversity of microfungi associated with insects in the tropics and clarify their taxonomy based on morphology and multigene phylogeny^14,15,19–24^.

## Results

### Molecular phylogeny

Table S1 comprises 39 taxa (including the seven newly collected taxa) analysed herein and their accession numbers. DNA sequence data of the new species have been submitted to GenBank. A concatenated sequence data-set was analyzed comprising 5003 characters with gaps (SSU: 971, LSU: 813, ITS: 667, TEF: 875, RPB1: 659, RPB2: 1018).

**Figure 1:**
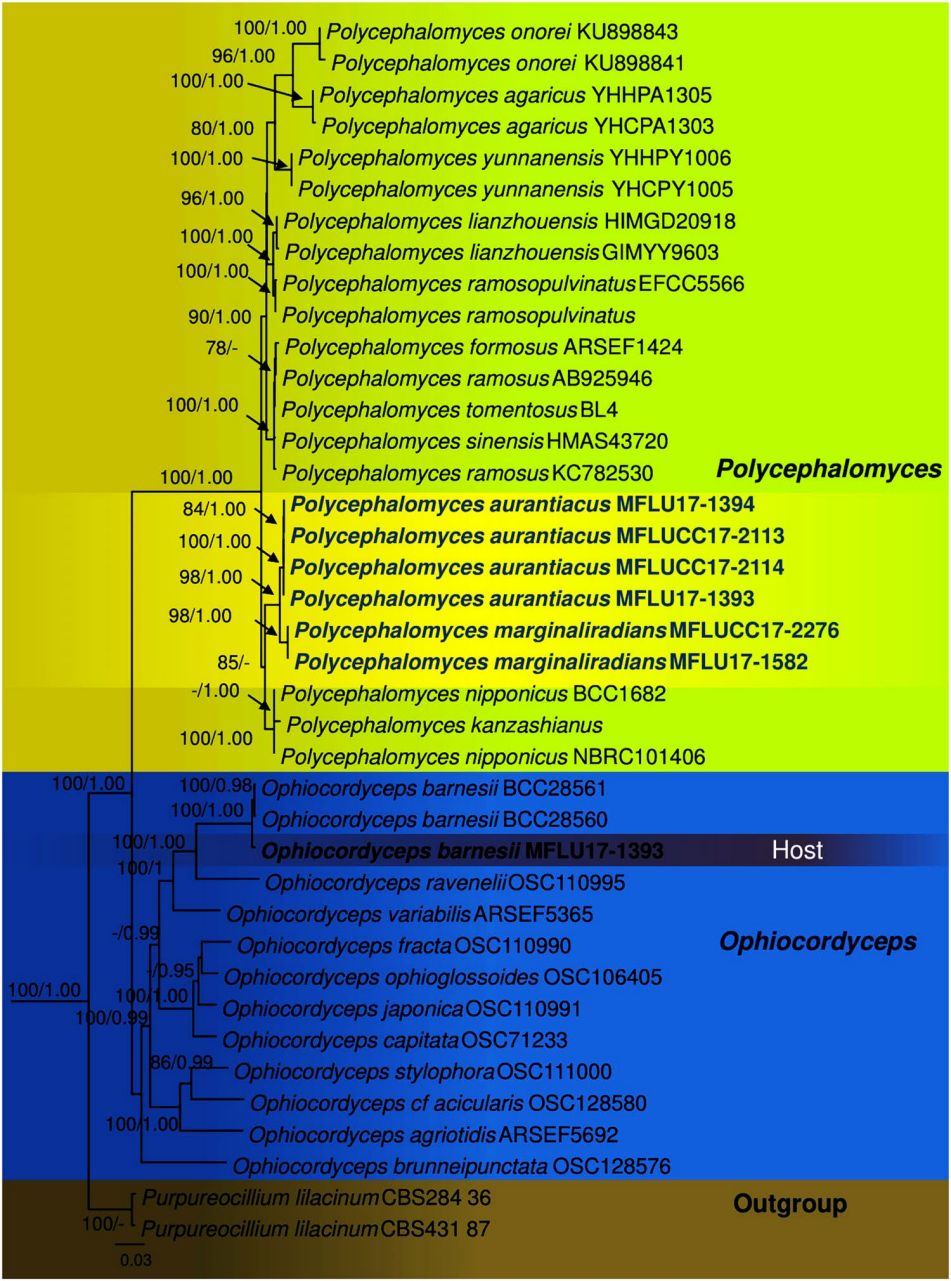
Phylogram of *Polycephalomyces aurantiacus*, *Polycephalomyces marginaliradians* and *Ophiocordyceps barnesii* generated from maximum likelihood analysis of ITS, SSU, LSU, RPB1, RPB2 and TEF1α sequence data. *Purpureocillium lilacinum* CBS 284.36 and *Purpureocillium lilacinum* CBS 431.87 were used as outgroup taxon. Maximum likelihood bootstrap values greater than 70% and Bayesian posterior probabilities over 0.9 are indicated above the nodes. The new species were indicated in blue. The host of *Polycephalomyces aurantiacus* is indicated in bold.

The RAxML analysis of the combined dataset yielded a best scoring tree (Fig. 1) with a final ML optimization likelihood value of −23008.064357. The matrix had 1750 distinct alignment patterns, with 39.76% of undetermined characters or gaps. Parameters for the GTR model of the concatenated data set were as follows: Estimated base frequencies; A = 0.236157, C = 0.276387, G = 0.278052, T = 0.209403; substitution rates AC = 1.217462, AG = 3.345152, AT = 0.776221, CG = 1.574418, CT = 6.177706, GT = 1.000; gamma distribution shape parameter α = 0.251124. The Bayesian analysis resulted in 20001 trees after 2000000 generations. The first 4000 trees, representing the burn-in phase of the analyses, were discarded, while the remaining 16001 trees were used for calculating posterior probabilities in the majority rule consensus tree.

The genus *Polycephalomyces* currently includes 15 species and only 11 species have available DNA sequence data in GenBank (Table S1), excluding the new taxa described in this study. Our multigene phylogenetic analyses herein reveal that our new taxa constitute a strongly supported monophyletic subclade and nested in between other *Polycephalomyces* species (Fig. 1). In particular it is noted that *Polycephalomyces aurantiacus* and *P. marginaliradians* share a close phylogenetic affinity to *P. nipponicus* and *P. kanzashianus* (Fig. 1).

In this paper, we illustrate a collection of *Ophiocordyceps barnesii*, which was parasitized by a *Polycephalomyces* species. Two new species of *Polycephalomyces*, one from *Ophiocordyceps barnesii*, and one from a Cossidae host are also introduced. A phylogenetic tree based on multigene sequence analyses for *Ophiocordyceps* (11 species) and *Polycephalomyces* (13 species), is also provided.

### Description of *Ophiocordyceps barnesii* (Thwaites) G.H. Sung, J.M. Sung, Hywel-Jones & Spatafora, in Sung, Hywel-Jones, Sung, Luangsa-ard, Shrestha & Spatafora, Stud. Mycol. 57: 40 (2007)

**Figure 2:**
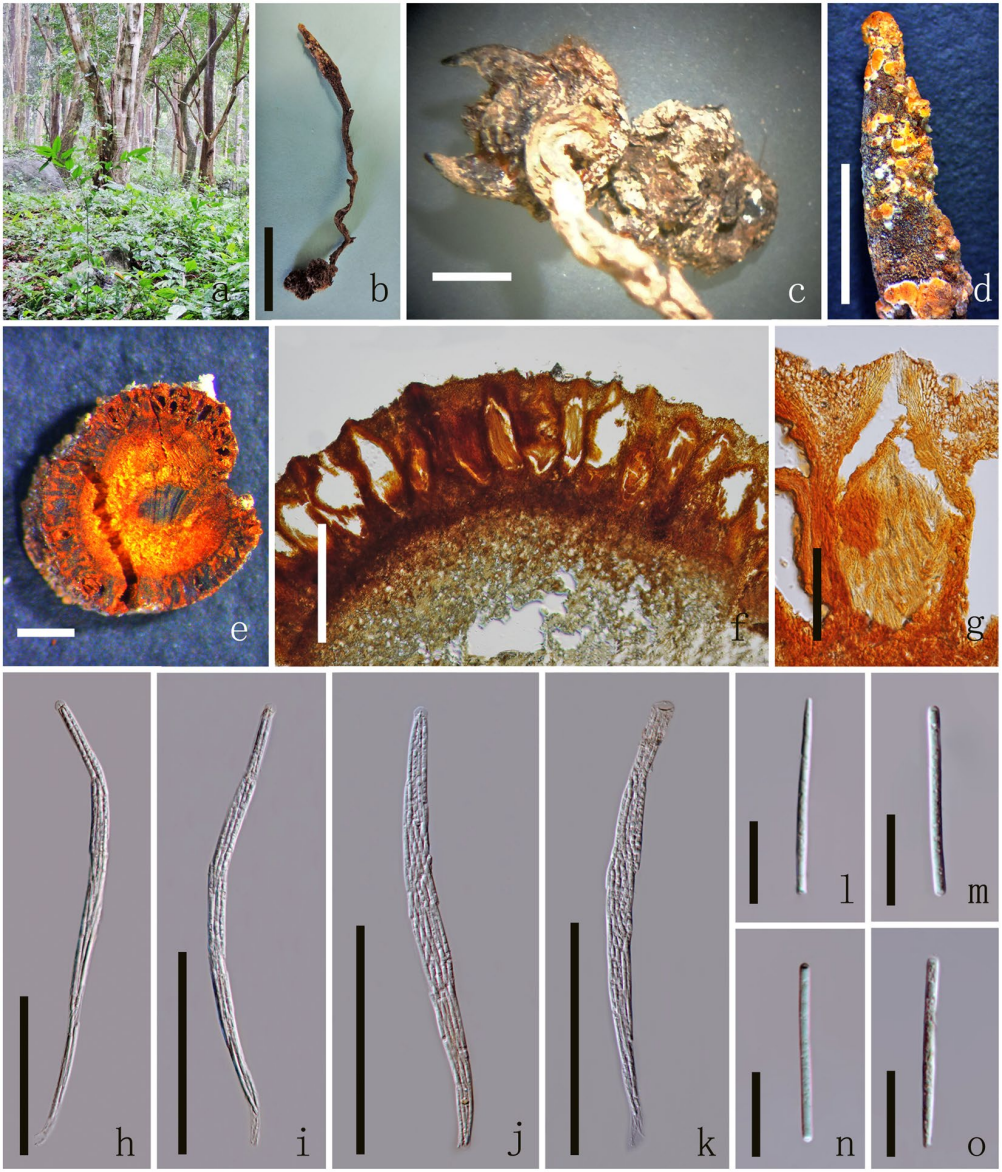
*Ophiocordyceps barnesii* MFLU 17-1393 (host). (**a**) Habitat. (**b**) Overview of the host and stromata. (**c**) Host. (**d**) Stroma. (**e**) Cross section of stroma. (**f**) Cross sections showing the immersed perithecia. (**g**) Perithecia. (**h**–**k**) Asci. (**l–o**) Secondary ascospores. Scale Bars: b = 5 cm, c = 5 mm, d = 2 cm, **e**, f = 500 µm, p = 200 µm, g–k = 100 µm, l–o, s = 20 µm, q = 10 µm, r = 5 µm.

Index Fungorum number: 504230; Facesoffungi number: FoF 03810 (Fig. 2).

*Parasitic* on larvae (Coleopteran), buried in the soil. Sexual morph: *Host* 2–2.5 long × 0.5–1 cm wide, brown to dark brown without hyphae on the surface. *Stromata* 13–20 long × 0.5–1 cm diam., mostly single, stipitate, unbranched or branched into 2 or 3 fertile head, arising from between the head and thorax of larva (fusiformis), dark-brown, fleshy, cylindrical, often flexuous or angularly crooked. *Stipe* 1–2 cm long, 2–3 mm diam., brown, with a fertile apex. *Fertile head* 2–5 cm long, 1–3.5 mm diam., single or branched more than 2, cylindrical, apically tapered, brown with orange mycelium cover on the surface. *Perithecia* 308–389 × 98–132 μm ($\bar{x}$ = 349 × 115 µm, n = 60), immersed, brown, elongated pyriform or flask-shaped, thick-walled. *Peridium* 12–21 µm ($\bar{x}$ = 16 µm, n = 60) wide, brown, *textura angularis* to *textura globulosa* to *textura prismatica*. *Asci* 195–229 × 6–9 μm ($\bar{x}$ = 212 × 7.5 µm, n = 90), 8-spored, hyaline, filiform, with a thin apex. *Apical cap* 3.1–4.2 × 4.1–5.4 μm ($\bar{x}$ = 3.6 × 4.7 µm, n = 60), with a small channel in the center. *Ascospores* 155–200 × 2.2–2.7 μm ($\bar{x}$ = 178 × 2.5 µm, n = 60), 3-septate, easily breaking into 4 part-spores, filiform, tapered at each end. *Secondary ascospores* 31.6–41.6 × 2.2–2.7 μm ($\bar{x}$ = 36.6 × 2.5 µm, n = 90) cylindrical, thickening at each end or tapered at one end, straight, hyaline, smooth-walled. Asexual morph: undetermined.

Notes: We collected *Ophiocordyceps barnesii* in this study which was colonized by an orange hyperparasite which we introduce below as *Polycephalomyces aurantiacus*. This species may be important in future industrial production of *Cordyceps* species, which are increasingly being produced because of their medicinal properties and biopesticides potential^25,26^. The specimen was deposited in MFLU Herbarium (MFLU 17-1393).

### Description of *Polycephalomyces aurantiacus* Y.P. Xiao, T.C. Wen & K.D. Hyde, *sp. nov*

**Figure 3:**
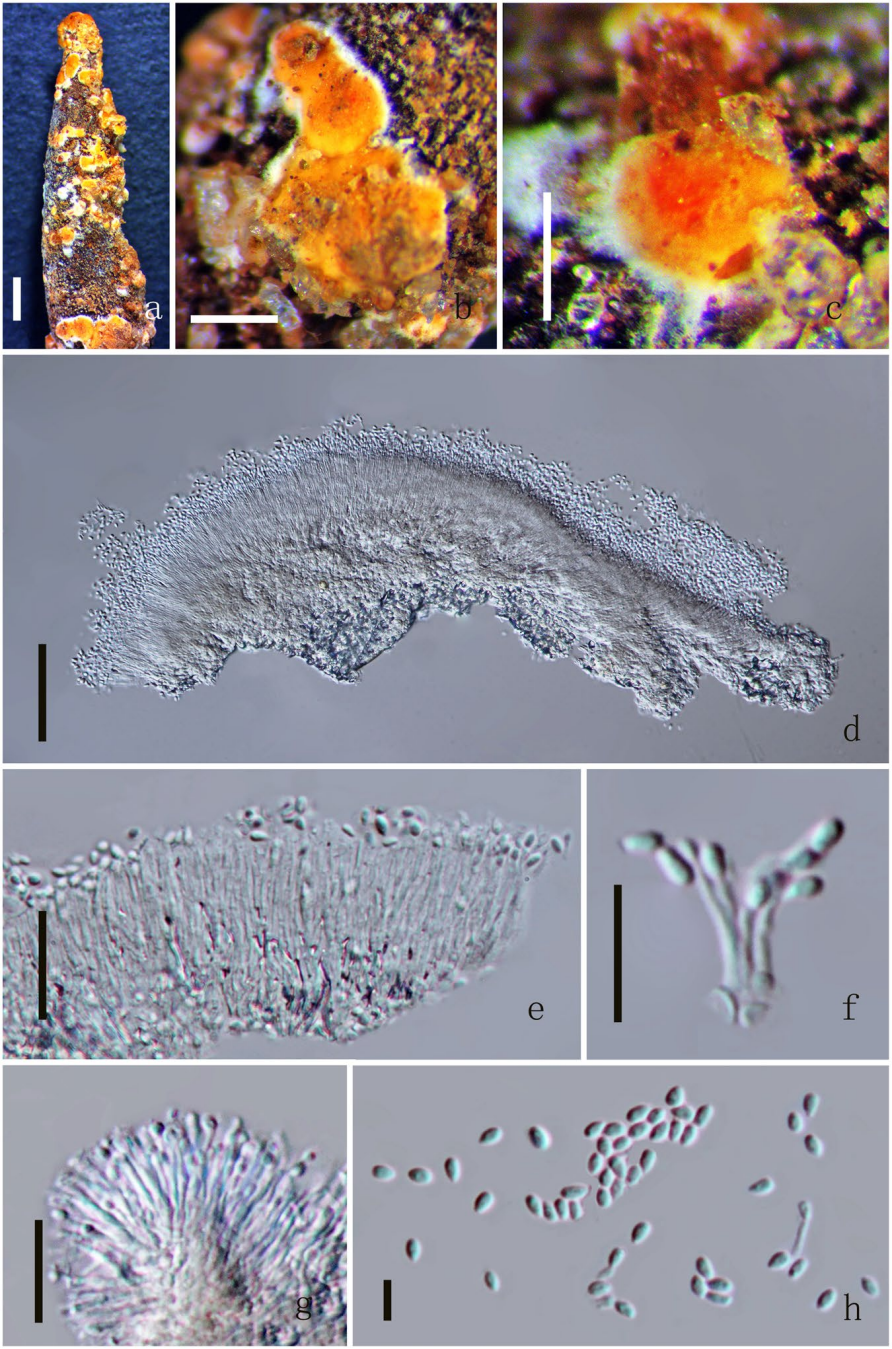
*Polycephalomyces aurantiacus* MFLU 17-1393. (**a**) Mycelium on the surface of stroma. (**b**,**c**) Conidiomata. (**d**) Section of conidioma. (**e–g**) Phialides. (**h**) Conidia. Scale Bars: a = 1000 µm, b = 500 µm, c = 200 µm, d = 50 µm, e = 20 µm, f–g = 10 µm, h = 5 µm.

**Figure 4:**
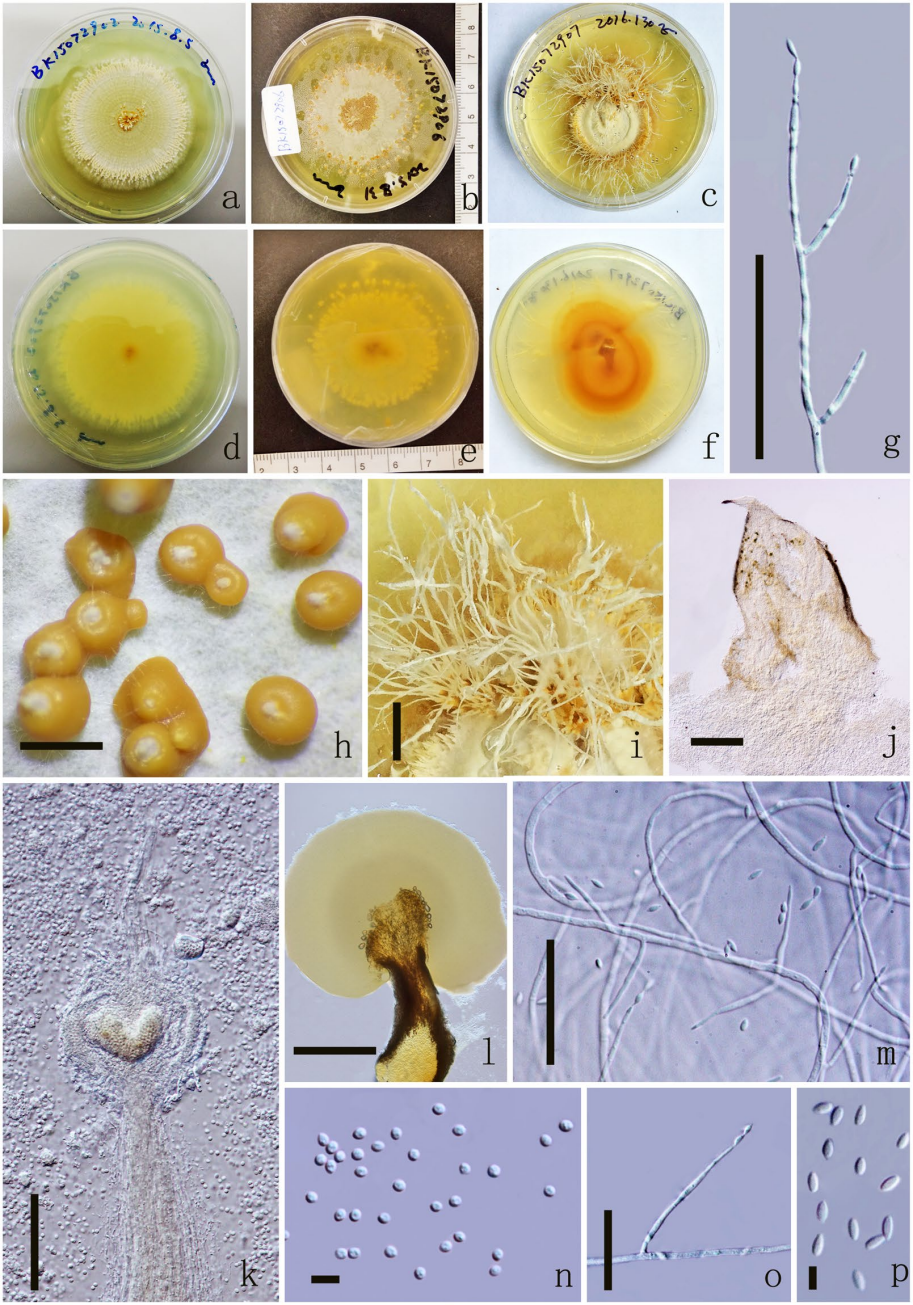
*Polycephalomyces aurantiacus* MFLUCC 17-2113. (**a–c**) Upper side of the culture. (**d**–**f**) Reverse side of the culture. (**g,o**) β-phialides. (**h–l**) Synnemata growing on PDA medium. (**m**) β-phialides with hyphae. (**n**) α-conidia. (**p**) β-conidia. Scale Bars: h = 1000 µm, i = 2000 µm, j = 200 µm, l = 500 µm, g, k, m = 50 µm, o = 20 µm, n, p = 5 µm.

Index Fungorum number: IF553936; Facesoffungi number: FoF 03811 (Figs 3, 4).

Etymology: The specific epithet refers to the color of conidia in mass in the specimen and colony.

Holotype: MFLU 17-1393

*Hyperparasite* on *Ophiocordyceps barnesii* (Ophiocordycipitaceae), buried in the soil. Sexual morph: undetermined. Asexual morph: *Synnemata* solitary or not solitary, arising from the fertile head of the stromata, flat-shaped, orange color. *Phialides* 9.9–14.3 × 0.7–1.4 μm ($\bar{x}$ = 12.1 × 1.1 µm, n = 90) hyaline. *Conidia* 2–2.6 × 1.4–2.1 μm ($\bar{x}$ = 2.3 × 1.8 µm, n = 90), oval to globose shape, hyaline, one-celled, smooth-walled, orange in mass.

*Colonies* on PDA medium, growing slowly, attaining 4 cm in 17 days at 25 °C, white, reverse yellow. *Synnemata* emerging after 30 days, solitary or not solitary, branched or unbranched, 1.3–2.2 μm long ($\bar{x}$ = 60), showing 1–2 radiating ring-like distributions. *Conidial masses* generating from the apex of the synnemata or covering the surface of the colony (Fig. 4). *Hyphae* hyaline, branched, smooth-walled, 0.3–50 mm ($\bar{x}$ = 20) wide. *Conidiophores* undetermined, not clear. *Phialides* existing in two types: α-and β-phialides. *α-phialides* 10.4–18.3 × 0.8–1.8 μm ($\bar{x}$ = 14.4 × 1.3 µm, n = 90) hyaline, narrow slender, smooth. *β-phialides* 22.9–64.2 × 1–1.5 μm ($\bar{x}$ = 43.6 × 1.3 µm, n = 90) solitary, growing from hyphae, narrow slender, catenateblasto conidia, smooth. *α-conidia* 1.8–2.2 × 1.4–1.9 μm ($\bar{x}$ = 2 × 1.7 µm, n = 90) globose to subglobose, occurring in the conidial mass on the agar or on the final portion of synnemata, one-celled, smooth-walled, yellow slimy in mass. *β-conidia* 3.2–3.9 × 1.4–1.8 μm ($\bar{x}$ = 3.5 × 1.6 µm, n = 90) fusiform, and produced on the surface mycelium of colony or on the top of the synnemata, one-celled, smooth-walled, hyaline, usually in chains on a phialide.

*Material examined*: THAILAND, Prachuap Khiri Khan. On dead larvae (Coleopteran), 29 July 2015, YuanPin Xiao, BK15072907 (MFLU 17-1393, holotype); BK15072902, BK15072906 (MFLU 17-1394, HKAS100693, paratypes); ex-type living cultures, MFLUCC 17-2113, MFLUCC 17-2114, MFLUCC 17-2115, KUMCC 17-0256, KUMCC 17-0257.

### Description of *Polycephalomyces marginaliradians* Y.P. Xiao, T.C. Wen & K.D. Hyde, *sp. nov*

**Figure 5:**
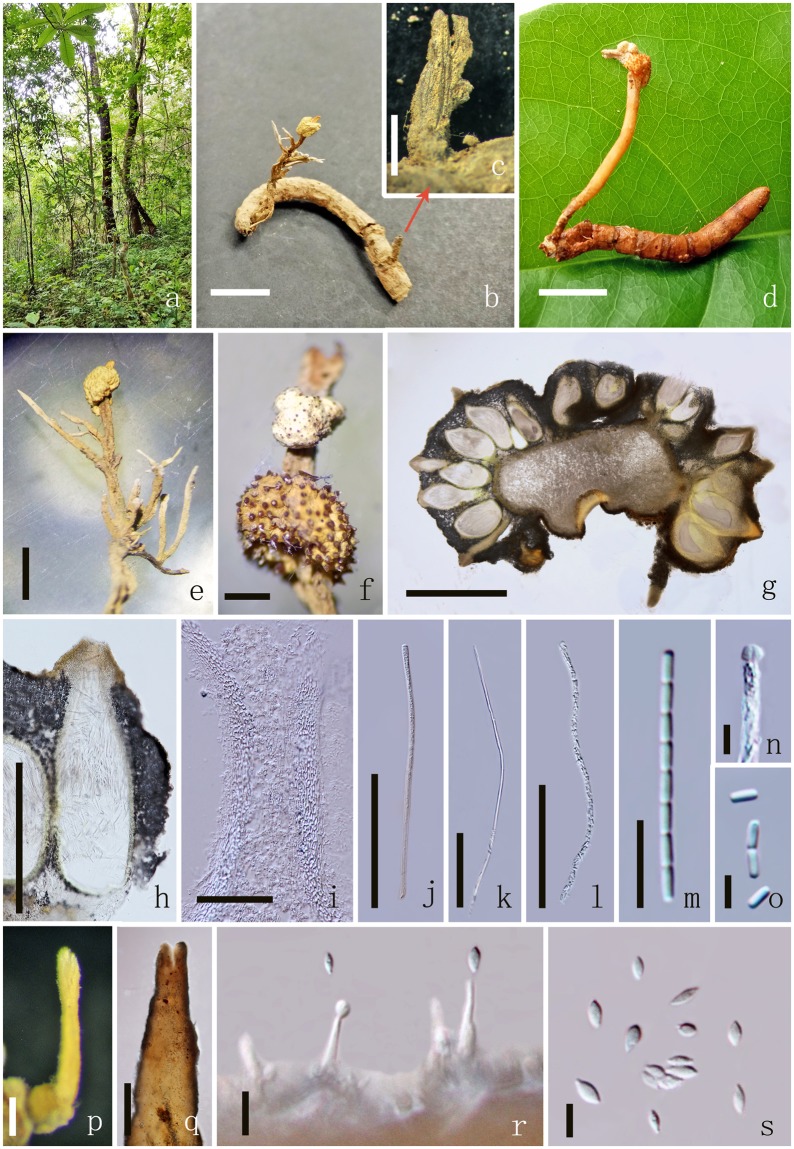
*Polycephalomyces marginaliradians* MFLU 17-1582. (**a**) Habitat. (**b,d**) Overview of the host and stromata. (**c**) Part of the stroma. (**e,f**) Stroma. (**g**) Cross sections showing the immersed perithecia. (**h**) Perithecia. (**j**–**l**) Asci. (**m**) Part of the ascospores. (**n**) Apical cap. (**o**) Secondary ascospores. (**p,q**) Synnemata. (**r**) Phialide. (**s**) Conidia. Scale Bars: b–d = 1 cm, e, f = 2000 µm, g = 1000 µm, h = 500 µm, p, q = 200 µm, j–l = 100 µm, i = 50 µm, m, r = 10 µm, n, o, s = 5 µm.

**Figure 6:**
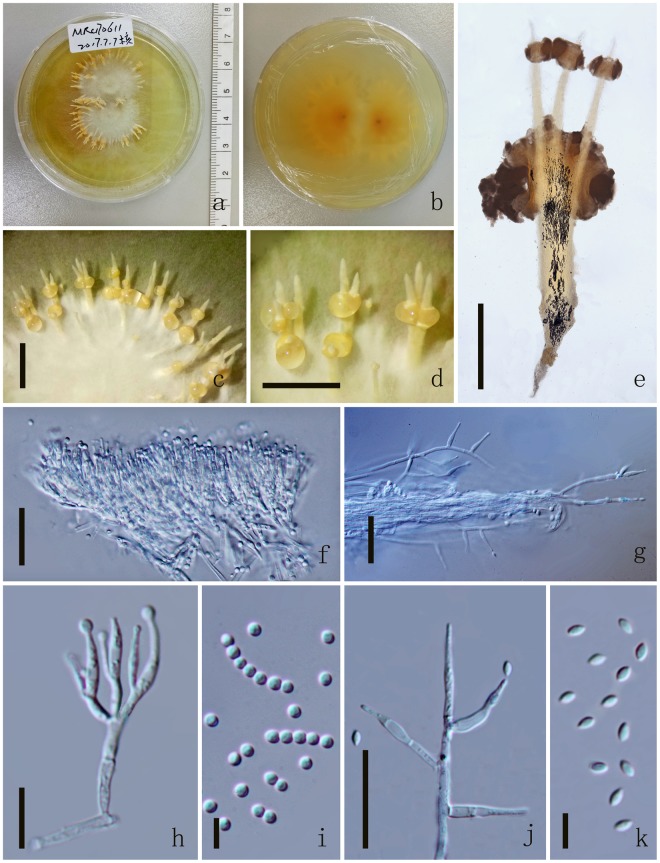
*Polycephalomyces marginaliradians* MFLUCC 17-2276. (**a**) Upper side of the culture. (**b**) Reverse side of the culture. (**c**,**d**) Synnemata growing on PDA medium. (**e**) Synnemata. (**f,h**) α-phialides. (**g,j**) β-phialides with hyphae. (**i**) α-conidia. (**k**) β-conidia. Scale Bars: c, d = 5000 µm, e = 1000 µm, f, g, j = 20 µm, h = 10 µm, i, k = 5 µm.

Index Fungorum number: IF553937; Facesoffungi number: FoF 03812 (Figs 5, 6).

Etymology: The specific epithet refers to the feature of the colonies on the culture.

Holotype: MFLU 17-1582

*Parasitic* on a Cossidae larva (Lepidoptera), buried in the soil. Sexual morph: *Thallus* within host. *Host* 3.2–3.5 long × 0.4–0.6 cm wide, yellow to brown, without hyphae on the surface. *Stromata* 3–3.5 long × 0.2–0.45 cm diam., mostly single, stipitate, cylindrical, unbranched or branched, arising from the head of larva, brown to yellow. *Stipe* 1–2 cm long, 2–3 mm diam., cylindrical, yellow to brown, with one or two lateral fertile head. *Fertile head* 0.40–0.42 cm long, 0.3–0.45 mm diam., capitate, lateral, globose to subglobose, pale yellow to yellow, with protruding ostiolar necks. *Ascomata* 676–803 × 246–328 μm ($\bar{x}$ = 739 × 287 µm, n = 60), immersed, yellow, flask-shaped, thick-walled. *Peridium* 11–19 µm ($\bar{x}$ = 15 µm, n = 60) wide, brown, textura angularis to textura globulosa to textura prismatica. *Asci* 459–556 × 3.1–4.3μm ($\bar{x}$ = 508 × 3.7 µm, n = 90), 8-spored, hyaline, filiform, with a thin apex. *Apical cap* 1.4–2.5 × 2.2–3.2 μm ($\bar{x}$ = 2 × 2.7 µm, n = 60), with a small channel in the center. *Ascospores* as long as the asci, easily breaking into part-spores, filiform. *Secondary ascospores* 3.2–4.2 × 1.3–1.7 μm ($\bar{x}$ = 3.8 × 1.5 µm, n = 90) cylindrical, straight, hyaline, smooth. Asexual morph: *Synnemata* solitary or not solitary, arising from the fertile head of the host, cylindrical, pale yellow. *Phialides* 11–14.4 × 1.2–1.8 μm ($\bar{x}$ = 12.7 × 1.5 µm, n = 90) hyaline. *Conidia* 3.6–4.9 × 1.8–2.5 μm ($\bar{x}$ = 4.2 × 2.1 µm, n = 90), fusiform, hyaline, one-celled, smooth-walled.

*Colonies* on PDA medium, circular, attaining 4 cm in 10 days at 25 °C, white, reverse yellow. *Synnemata* emerging after 14 days in the margin of the colony, single or branched into 2 or 3 branched, 3200.8–4566.3 × 142.9–661.8 μm ($\bar{x}$ = 3883.5 × 402.3 µm, n = 30), showing 1–2 radiating ring-like distributions. *Conidial masses* generating from the middle of the synnemata or covering the surface of the colony, pale yellow to yellow, with hyaline to pale yellow exucate. *Hyphae* hyaline, branched, smooth-walled, 1.8–2.7 μm ($\bar{x}$ = 2.2) wide. *Conidiophores* undetermined, not clear. *Phialides* existing in two types: α-and β-phialides. *α-phialides* 11–14.4 × 1.2–1.8 μm ($\bar{x}$ = 12.7 × 1.5 µm, n = 90), hyaline, smooth, elongated lageniform,caespitose, palisade-like, crowed, monoverticillate, mostly branched into 2 phialides, 3 branched on one metula. *β-phialides* 12.8–23.9 × 1.8–2.7 μm ($\bar{x}$ = 18.3 × 2.2 µm, n = 90), hyaline, smooth, solitary, growing from hyphae, narrow slender to narrow lageniform, with or without metula at the base. *α-conidia* 1.9–2.6 μm ($\bar{x}$ = 2.3 µm, n = 90) diam, globose, catenate, occurring in the conidial mass on the middle of synnemata, one-celled, smooth-walled, pale yellow slimy in mass. *β-conidia* 3.1–3.9 × 1.6–2.1 μm ($\bar{x}$ = 3.5 × 1.8 µm, n = 90) fusiform, and produced on the surface mycelium of colony or on the branch of the synnemata, one-celled, smooth-walled, hyaline.

*Material examined*: THAILAND, Chiang Mai, The Mushroom Research Center. On dead Cossidae larvae (Lepidoptera), 11 June 2017, Yuan Pin Xiao, MRC170611 (MFLU 17-1582, holotype); CM48 (MFLU 17-1583 MFLU 17-1584, HKAS100694, paratypes); ex-type living cultures, MFLUCC 17-2276, MFLUCC 17-2277, MFLUCC 17-2278, KUMCC 17-0258, KUMCC 17-0259.

## Discussion

Studies based on morphology and DNA sequence analyses have provided insights into the phylogeny of *Polycephalomyces* to resolve generic delimitation. Species of this genus are commonly known to exhibit a parasitic mode of life on insects and other fungi^2,5,12^. Our fungal diversity studies on entomophagous fungi have led to the discovery of two species, new to science, which we accommodate in *Polycephalomyces*. Molecular data also reveals that our new genus belongs to the family Ophiocordycipitaceae as circumscribed by Matočec *et al*.^12^. Species which exist in their sexual state display morphs such as fertile, capitulate, globose, tuberiform to pulvinate stromata and immersed, elongated pyriform perithecia while the asexual morphs occur as branched or unbranched synnemata, ending up with clavate to spherically flared, hymeniform aggregations of conidiophores, and produce large masses of conidia united in collective globular mucus^12^. To date, six species, including *P. ramosus*^16,17^, *P. sinensis*^6,18^, and *P. agaricus*^9^ are considered as parasites of entomogenous fungi, while six species are recorded as entomogenous^6,7,9,11^. Some species such as *P. lianzhouensis* and *P. yunnanensis* colonise both entomogenous fungi and insects^7,8^. Because of their economic importance, species of this genus have been the subject for various research. The most recent new species introduced is *P. yunnanensis* and multigene phylogeny reveals a close relationship to *P. formosus*, *P. ramosopulvinatus* and *P. sinensis* based on 5-loci (nr*SSU*, nr*LSU*, *tef-1α*, *rpb1* and *rpb2*) phylogenetic analyses^8^.

Our taxonomic investigations herein reveal two new species of *Polycephalomyces*, *P. aurantiacus* and *P. marginaliradians*. Our morphological examination suggests that both species fit clearly within the generic concept of *Polycephalomyces* and both species produce two types of conidia. However, they exhibit different mode of life and there are sufficent morphological differences that can justify their segregation into two species. These two new species similar to *P. agaricus*, *P. formosus*, *P. ponerae*, *P. sinensis*, *P. ramosus*, and *P. yunnanensis* have produce two types of conidia, while *P. ditmarii*, *P. lianzhouensis*, *P. paludosus* and *P. tomentosus* have only one type of conidia. *Polycephalomyces aurantiacus* and *P. marginaliradians* have two types of phialides, while *P. formosus* and *P. sinensis* have only one type of phialide. *Polycephalomyces ponerae* also differs from *P. aurantiacus* and *P. marginaliradians* by producing *Akanthomyces*-like β-phialides and parasitic on ant (*Ponera Latreille*). *Polycephalomyces agaricus* differs from *P. aurantiacus* and *P. marginaliradians* by producting agaric shaped synnemata and parasitic on *Ophiocordyceps sp*. *Polycephalomyces yunnanensis* is distinct from *P. aurantiacus* and *P. marginaliradians* as it produces longer α-conidia fusiform, catenate or clump together β-conidia and parasitic on *O. nutans*. *Polycephalomyces ramosus* differs from *P. aurantiacus* and *P. marginaliradians* in having longer synnemata, shorter β-phialides and parasitic on *Hirsutella guignardii*. *Polycephalomyces aurantiacus* is distinct from *P. marginaliradians* as it is parasitic on the fungus *Ophiocordyceps barnesii* and produces longer synnemata, shorter β-phialides and catenate α-conidia, wheras *P. marginaliradians* is parasitic on insect and produces shorter synnemata, longer β-phialides and catenate β-conidia (further morphological differences are outlined in Table S2).

Phylogeny based on our concatenated dataset recovered also support that our two new species belong to *Polycephalomyces* and are distinct from each other (Fig. 1). A close relationship is observed between the two species, but both constitute independent and strongly supported monophyletic subclades indicative of two phylogenetically distinct speces. To further compare our two species, we delved in pairwise nucleotide sequence comparison and noted sufficient differences to justify them as independent taxa^27^. ITS pairwise nucleotide sequence comparison between *P. aurantiacus* and *P. marginaliradians* revealed striking differences in 15 base pairs that justifies that both are different from each other and hence can be considered as two distinct species. There are also 6, 21, 4, 15 and 13 differences in the nr*SSU*, nr*LSU*, *tef-1α*, *rpb*1, and *rpb2* DNA sequence data respectively. Two species not considered in our phylogenetic sampling are *P. ditmarii* and *P. paludosus* due to the unavailability of sequence data. However these two are different from our new species with respect to one type of conidia occurring on its natural substrate and under cultural conditions^28,29^. The hosts from which our new species have been recovered are also different. *Polycephalomyces ponerae* was not considered in our phylogenetic sampling as the DNA (ITS) sequence is too short, ambiguous and did not align well with other species. However, *Polycephalomyces ponerae* is morphologically different from our new species with respect to *Akanthomyces*-like β-phialides and parasitic on ant (*Ponera Latreille*). Further morphological differences among species are detailed in Tables S2 and S3.

Our multigene phylogeny derived herein also provides robust and well-resolved intergeneric relationships between *Polycephalomyces* and *Ophiocordyceps*. Members of both genera are clearly distinct from each other and we managed to successfully identify and sequence *Ophiocordyceps barnesii*, the host from which *P. aurantiacus* was isolated. Further interspecies taxonomic relationships are also elucidated in our molecular phylogeny. All *Polycephalomyces* species currently analysed constitute a strongly supported monophyletic lineage (Fig. 1), which corroborates with previous taxonomic schemes^5,12^. In particular, a robust relationship in observed between *P. onorei* and *P. agaricus* sharing *P. yunnanensis* as sister taxa. These three species are also markedly different in terms of morphological characters. *Polycephalomyces yunnanensis* is clearly distinct from *P. onorei* and *P. agaricus* in terms of being parasitic on *Ophiocordyceps nutans* (Pat.) G.H. Sung, J.M. Sung, Hywel-Jones & Spatafora, longer synnemata, cylindrical to subulate α-phialides and subglobose or ellipsoidal α-conidia^8,10^. *Polycephalomyces ramosopulvinatus* is closely related to *P. lianzhouensis*, but each species is positioned in different well-supported subclades and hence merit species status. *Polycephalomyces ramosopulvinatus* is also different from *P. lianzhouensis* with respect to being parasitic on nymph of Cicada and characterised by a long stipe and pseudo-immersed, pyriform perithecia. While phylogeny resolves our new species into well-segregated subclades, we note that relationships of *P. formosus, P. tomentosus, P. ramosus* and *P. sinensis* are still obscure and the concatenated dataset used herein did not provide adequate species resolution. A similar phylogenetic scenario is observed for *P. nipponicus* and *P. kazanshianus*. Whether these species are conspecific warrants further taxonomic investigations. The latter two species do share some morphological resemblances to *P. marginaliradians* especially with respect to the yellow cylindrical stipe with capitate lateral fertile part (known from their sexual morph). However, *P. marginaliradians* differs in having a capitate stromata with stipe, smaller perithecia and parasitic on Cossidae, while *P. nipponicus* and *P. kanzashianus* have polycephalous stromata and parasitic on Cicadidae. Meanwhile, *P. onorei* and *P. ramosopulvinatus* are distinct from *P. marginaliradians* by producing bigger perithecia and parasitic on caterpillar (*Arctinae*) and nymph of Cicada respectively. Phylogenies retrieved herein also support them as separate taxonomic entities.

Key to the species of the genus *Polycephalomyces*

1. Synnemata arising from fungi or insect or culture…………………………2

1. Stromata arising from insect…………………………13

2. Two types of conidia absent in nature or culture…………………………3

2. One type of conidia absent in nature and culture…………………………10

3. Synnemata agaric-shaped…………………………***P. agaricus***

3. Synnemata other shaped…………………………4

4. Two types of phialides absent…………………………5

4. Only one type of phialides absent…………………………9

5. α-conidia globose to subglobose (1.4–3.2 × 1.2–2.2) μm…………………………6

5. α-conidia ovoid (2.4–3.2 × 1.6–2.4) μm…………………………***P. ramosus***

6. β-phialides *Akanthomyces*-like, inflated at base, slenderneck at top…………………………***P. ponerae***

6. β-phialides lanceolate or narrowly lageniform or subulate…………………………7

7. β-phialides lanceolate or narrowly lageniform, 22.9–64.2 μm length…………………………***P. aurantiacus***

7. β-phialides narrowly lageniform or subulate, 7–30 μm length…………………………8

8.α-conidia subglobose, not catenate, β-conidia fusiform, catenate…………………………***P. marginaliradians***

8. α-conidia globose, catenate, β-conidia fusiform, not catenate…………………………***P. yunnanensis***

9. Phialides lanceolate or narrowly lageniform, 12.5–66 μm length…………………………***P. sinensis***

9. Phialides cylindrical, subulate, 10–15 μm length…………………………***P. formosus***

10. Host is insect…………………………11

10. Host is myxomycetes…………………………***P. tomentosus***

11. Conidia globose to subglobose or cylindrical…………………………12

11. Conidia obovoid, covered by a mucus, agglutinating…………………………***P. paludosus***

12. Conidia globose to subglobose, 2.2–3.4 × 1.3–1.6um…………………………***P. ditmarii***

12. Conidia subglobose to cylindrical, 5–7 × 1.3–1.6um…………………………***P. lianzhouensis***

13. Host is Cicada or nymph of Cicada…………………………14

13. Host is neither Cicada nor nymph of Cicada…………………………16

14. Stipe less than 80 mm…………………………15

14. Stipe more than 90 mm…………………………***P. ramosopulvinatus***

15. Perithecia flask-shaped, 900–1050 × 270–300 um.…………………………***P. kanzashianus***

15. Perithecia flask-shaped or ovioid, 800–950 × 300–370 um…………………………***P. nipponicus***

16. Stromata numerous…………………………17

16. Stromata single…………………………***P. marginaliradians***

17. Fertile part narrowly ovoid 355–473 × 158–197 μm…………………………***P. lianzhouensis***

17. Fertile part pyriform, 854–950 × 330–395 um…………………………***P. onorei***

## Materials and Methods

### Collection, isolation, and morphology study

Four fresh specimens were collected from southern Thailand (Prachuap Khiri Khan Province), and two from northern Thailand in the soil. The specimens were noted and photographed in the field and transported to the laboratory individually in plastic boxes and stored at 4 °C until examined. Strains were isolated from single spore isolation from both stomata and synnemata following the protocol described in Chomnunti *et al*.^30^.Cultures were incubated at 18 °C for 14–25 days on potato extract agar (PDA) as outlined by Vijaykrishna *et al*.^31^. Herbarium material is deposited at MFLU herbarium and HKAS herbarium and Facesoffungi numbers and Index Fungorum numbers are provided as in Jayasiri *et al*.^32^ and Index Fungorum^33^. New species are based on recommendations outlined by Jeewon & Hyde^27^.

### DNA extraction, PCR amplification and determination of DNA sequences

DNA was extracted from both dried specimens and cultures by using E.Z.N.A.TM Fungal DNA MiniKit (Omega Biotech, CA, USA) according to the manufacturer’s protocols. The primers used in PCR amplification were (Table S4); ITS4/ITS5 for internal transcribed spacer gene region (ITS)^34^, NS1/NS4 for partial small subunit ribosomal RNA gene region (SSU)^34^, LROR/LR5 for partial large subunit rDNA gene region (LSU)^35^. 983 F/2218 R for partial translation elongation factor 1-alpha gene region (TEF-1α)^36^, CRPB1A/RPB1Cr for partial RNA polymerase II largest subunit gene region (RPB1)^37^. RPB2-5F/RPB2-5R for partial RNA polymerase II second largest subunit gene region (RPB2)^37^. PCR amplifications were conducted as outlined by Jeewon *et al*.^38,39^ and PCR products were sequenced by GenScript Biotechnology Co., Nanjing, China.

### Phylogenetic analyses

All reference sequences were obtained from GenBank based on previously published data (Table S1). MAFFT v.7^40^ (http://mafft.cbrc.jp/alignment/server/) was used to align combined datasets of ITS, SSU, LSU, TEF1α and RPB1. BioEdit^41^ was used to check alignment manually. Gaps were treated as missing data. Two strains of *Perennicordyceps prolifica* (Kobayasi) Matočec & I. Kušan, in Matočec *et al*.^12^ were selected as outgroup taxa.

ML trees were estimated by using the software RAxML 7.2.8 Black Box^42,43^ in the CIPRES Science Gateway platform^44^. MrModeltest v.2.3^45^ was used to determine the best-fit model of evolution for Bayesian analyses. MrBayes v.3.1.2^46^ was used to evaluate posterior probabilities (PP)^47,48^ by Markov Chain Monte Carlo sampling (BMCMC). Six simultaneous Markov chains were run for 2,000,000 generations and trees were sampled every 100th generation and 20,000 trees were obtained. The first 20% of trees were discarded, which representing the burn-in phase of the analyses, while the remaining trees were used for calculation posterior probabilities in the majority rule consensus tree (critical values for the topological convergence diagnostic is 0.01). Pylogenetic trees were also constructed based on parsimony analyses as detailed by Cai *et al*.^49^ and Jeewon *et al*.^50,51^. Trees were figured in FigTree v1.4.0 program^52^. Bayesian Posterior Probabilities (BYPP) equal to or great than 0.95 were given^53–63^ below each node (Fig. 1).

## Electronic supplementary material

Supplementary Table

## References

[1] Kobayasi Y (1941). The genus Cordyceps and its allies. Sci Rep Tokyo BunrikaDaigaku, Sec B..

[2] Wijayawardene (2017). Notes for genera: Ascomycota. Fungal Diversity..

[3] Wijayawardene (2018). Outline of Ascomycota – 2017. Fungal Diversity..

[4] Massee G (1895). A revision of the genus Cordyceps. Annals bot-london..

[5] Kepler R (2013). The phylogenetic placement of hypocrealean insect pathogens in the genus Polycephalomyces: an application of One Fungus One Name. Fungal biol-uk..

[6] Wang WJ (2012). Molecular and morphological studies of *Paecilomyces sinensis* reveal a new clade in clavicipitaceous fungi and its new systematic position. Syst Biodivers..

[7] Wang L, Li HH, Chen YQ, Zhang WM, Qu LH (2014). *Polycephalomyces lianzhouensis* sp. nov., a new species, co-occurs with *Ophiocordyceps crinalis*. Mycol prog..

[8] Wang YB (2015). *Polycephalomyces yunnanensis* (Hypocreales), a new species of Polycephalomyces parasitizing Ophiocordyceps nutans and stink bugs (hemipteran adults). Phytotaxa..

[9] Wang YB (2015). *Polycephalomyces agaricus*, a new hyperparasite of Ophiocordyceps sp. infecting melolonthid larvae in southwestern China. Mycol Prog..

[10] Crous PW (2017). Fungal Planet description sheets: 558–624. Persoonia..

[11] Liang Z, Chen W, Liang J, Han Y, Zou X (2016). Phenotypic polymorphism of the synnematous entomogenous fungi in an ant nest of Ponera I. Mycosystema.

[12] Matočec N, Kušan I, Ozimec R (2014). The genus Polycephalomyces (Hypocreales) in the frame of monitoring Veternica cave (Croatia) with a new segregate genus Perennicordyceps. Ascomycete. org..

[13] Ban S, Sakane T, Toyama K, Nakagiri A (2009). Teleomorph–anamorph relationships and reclassification of *Cordyceps cuboidea* and its allied species. Mycoscience.

[14] Maharachchikumbura SSN (2015). Towards a natural classification and backbone tree for Sordariomycetes. Fungal Divers..

[15] Maharachchikumbura SSN (2016). Families of Sordariomycetes. Fungal Divers..

[16] Seifert K (1985). A monograph of Stilbella and some allied Hyphomycetes. Stud in Mycol..

[17] Bischoff JF, Sullivan RF, Hywel-Jones NL, White JF (2003). Resurrection of Blistum tomentosum and its exclusion from Polycephalomyces (Hyphomycetes, Deuteromycota) based on 28S rDNA sequence data. Mycotaxon..

[18] Chen, Q.T., Xiao, S.R. & Shi, Z.Y. *Paecilomyces sinensis* sp. nov. and its connection with *Cordyceps sinensis*. *Acta Mycologica Sinica*. 24–28 (1984).

[19] Wen TC, Xiao YP, Li WJ, Kang JC, Hyde KD (2014). Systematic analyses of *Ophiocordyceps ramosissimum* sp. nov., a new species from a larvae of Hepialidae in China. Phytotaxa..

[20] Wen T (2015). *Metacordyceps shibinensis* sp. nov. from larvae of Lepidoptera in Guizhou Province, southwest China. Phytotaxa..

[21] Wen TC, Xiao YP, Zha LS, Hyde KD, Kang JC (2016). Multigene phylogeny and morphology reveal a new species, *Ophiocordyceps tettigonia*, from Guizhou Province, China. Phytotaxa..

[22] Wen TC (2017). Multigene phylogeny and morphology reveal that the Chinese medicinal mushroom ‘*Cordyceps gunnii*’is *Metacordyceps neogunnii* sp. nov. Phytotaxa..

[23] Li GJ (2016). Fungal diversity notes 253–366: taxonomic and phylogenetic contributions to fungal taxa. Fungal Divers..

[24] Tibpromma S (2017). Fungal diversity notes 491–602: taxonomic and phylogenetic contributions to fungal taxa. Fungal Divers..

[25] Cock MJ, Allard GB (2013). Observations on white grubs affecting sugar cane at the Juba Sugar Project, South-Western Somalia, in the 1980s, and implications for their management. Insects..

[26] Han H, Du L, An T (2015). Main Wild Medical Fungi and Their Functions in Eastern Forest Region of Zhongtiao Mountain in Shanxi Province. Agricultural Science & Technology..

[27] Jeewon R, Hyde KD (2016). Establishing species boundaries and new taxa among fungi: recommendations to resolve taxonomic ambiguities. Mycosphere..

[28] Van Vooren N, Audibert C (2005). Révision du complexe «*Cordyceps sphecocephala*». 1re partie: les guêpes végétales. B mens Soc linn Lyon..

[29] Mains EB (1948). Entomogenous fungi. Mycologia..

[30] Chomnunti P (2014). The sooty moulds. Fungal Divers..

[31] Vijaykrishna D (2004). *Pleurostomophora*, an anamorph of *Pleurostoma* (Calosphaeriales), a new anamorph genus morphologically similar to *Phialophora*. Stud Mycol.

[32] Jayasiri SC (2015). The Faces of Fungi database: fungal names linked with morphology, phylogeny and human impacts. Fungal Divers..

[33] Index Fungorum. www.indexfungorum.org 2017 (2017).

[34] White TJ, Bruns T, Lee S, Taylor JW (1990). Amplification and direct sequencing of fungal ribosomal RNA genes for phylogenetics. PCR Protocols: a guide to methods and applications..

[35] Vilgalys R, Hester M (1990). Rapid genetic identification and mapping of enzymatically amplified ribosomal DNA from several Cryptococcus species. J Bacteriol..

[36] Sung GH, Sung JM, Hywel-Jones NL, Spatafora JW (2007). A multi-gene phylogeny of Clavicipitaceae (Ascomycota, Fungi): identification of localized incongruence using a combinational bootstrap approach. Mol Phylogenet Evol..

[37] Castlebury LA, Rossman AY, Sung GH, Hyten AS, Spatafora JW (2004). Multigene phylogeny reveals new lineage for Stachybotrys chartarum, the indoor air fungus. Mycol Res.

[38] Jeewon R, Cai L, Liew ECY, Zhang K, Hyde KD (2003). *Dyrithiopsis lakefuxianensis* gen et sp. nov. from Fuxian Lake, Yunnan, China and notes on the taxonomic confusion surrounding *Dyrithium*. Mycologia.

[39] Jeewon R (2017). Nomenclatural and identification pitfalls of endophytic mycota based on DNA sequence analyses of ribosomal and protein genes phylogenetic markers: A taxonomic dead end?. Mycosphere.

[40] Katoh K, Standley DM (2013). MAFFT multiple sequence alignment software version 7: improvements in performance and usability. Mol Biol Evol..

[41] Hall T (2011). BioEdit: an important software for molecular biology. GERF Bull. Biosci..

[42] Stamatakis A (2006). RAxML-VI-HPC: maximum likelihood-based phylogenetic analyses with thousands of taxa and mixed models. Bioinformatics..

[43] Stamatakis A, Hoover P, Rougemont J (2008). A rapid bootstrap algorithm for the RAxML web servers. Syst Biol..

[44] Miller, M.A., Pfeiffer, W. & Schwartz, T. Creating the CIPRES Science Gateway for inference of large phylogenetic trees. *Gateway Computing Environments Workshop (GCE)*. 1–8 (2010).

[45] Nylander, J. A. MrAIC [Internet] Available from: http://www.abc.se/∼nylander/. program distributed by the author (2004).

[46] Ronquist F, Huelsenbeck JP (2003). MrBayes 3: Bayesian phylogenetic inference under mixed models. Bioinformatics..

[47] Rannala B, Yang Z (1996). Probability distribution of molecular evolutionary trees: a new method of phylogenetic inference. J Mol Evol..

[48] Zhaxybayeva O, Gogarten JP (2002). Bootstrap, Bayesian probability and maximum likelihood mapping: exploring new tools for comparative genome analyses. BMC Genomics..

[49] Cai L, Jeewon R, Hyde KD (2005). Phylogenetic evaluation and taxonomic revision of *Schizothecium* based on ribosomal DNA and protein coding genes. Fungal Divers.

[50] Jeewon R, Liew ECY, Hyde KD (2002). Phylogenetic relationships of *Pestalotiopsis* and allied genera inferred from ribosomal DNA sequences and morphological characters. Mol Phylogenet Evol.

[51] Jeewon R, Liew ECY, Simpson JA, Hodgkiss IJ, Hyde KD (2003). Phylogenetic significance of morphological characters in the taxonomy of *Pestalotiopsis* species. Mol Phylogenet Evol.

[52] Rambaut, A. Figtree 1.4.0. http://tree.bio.ed.ac.uk/software/figtree/ (18 August 2014, date last accessed) (2012).

[53] Kepler RM (2012). New teleomorph combinations in the entomopathogenic genus Metacordyceps. Mycologia..

[54] Luangsa-Ard JJ, Ridkaew R, Mongkolsamrit S, Tasanathai K, Hywel-Jones NL (2010). *Ophiocordyceps barnesii* and its relationship to other melolonthid pathogens with dark stromata. Fungal Biol..

[55] Spatafora JW, Sung GH, Sung JM, Hywel-Jones NL, White JF (2007). Phylogenetic evidence for an animal pathogen origin of ergot and the grass endophytes. Mol Ecol..

[56] Nikoh N, Fukatsu T (2000). Interkingdom host jumping underground: phylogenetic analysis of entomoparasitic fungi of the genus Cordyceps. Mol Biol Evol..

[57] Zhang WM, Wang L, Tao MH, Chen YQ, Qu LH (2007). Two species of Cordyceps simultaneously parasitic on a larva of Lepidoptera. Mycosystema..

[58] Schoch CL (2012). Nuclear ribosomal internal transcribed spacer (ITS) region as a universal DNA barcode marker for Fungi. P Natl Acad Sci USA.

[59] Kobayasi Y, Shimizu D (1982). Cordyceps species from Japan 4. Bulletin of the National Science Museum Tokyo..

[60] Kobayasi Y (1939). On the genus Cordyceps and its allies on cicadae from Japan. Bulletin of the Biogeographical Society of Japan..

[61] Sung GH, Spatafora JW, Zare R, Hodge KT, Gams W (2001). A revision of Verticillium sect. Prostrata. II. Phylogenetic analyses of SSU and LSU nuclear rDNA sequences from anamorphs and teleomorphs of the Clavicipitaceae. Nova Hedwigia..

[62] Luangsa-ard JJ, Hywel-Jones NL, Samson RA (2004). The polyphyletic nature of *Paecilomyces sensu lato* based on 18S-generated rDNA phylogeny. Mycologia.

[63] Luangsa-ard J (2011). *Purpureocillium*, a new genus for the medically important *Paecilomyces lilacinus*. FEMS Microbiol Lett.

