# Supplementary material for: Multigene phylogenetics of Polycephalomyces (Ophiocordycipitaceae, Hypocreales), with two new species from Thailand
Source: Sci Rep. 2018 Dec 27;8:18087. doi: 10.1038/s41598-018-36792-4 (PMC6308235; doi:10.1038/s41598-018-36792-4)
Supplement: Supplementary file 1 — Supplementary Table [file 41598_2018_36792_MOESM1_ESM.pdf]

Multigene phylogenetics of *Polycephalomyces* (Ophiocordycipitaceae, Hypocreales), with two new species from Thailand

Yuan-Pin Xiao[1,2,3], Ting-Chi Wen[1*], Sinang Hongsanan[2], Rajesh Jeewon[5], J. Jennifer Luangsa-ard[4], Siraprapa Brooks[3], Dhanushka Nadeeshan Wanasinghe[2,3], Feng-Yao Long[1] & Kevin D. Hyde[2,3]

[1]Engineering Research Center of Southwest Bio-Pharmaceutical Resources, Ministry of Education, Guizhou University, Guiyang, Guizhou Province 550025, China

[2]Center of Excellence in Fungal Research, Mae Fah Luang University, Chiang Rai 57100, Thailand

[3]School of Science, Mae Fah Luang University, Chiang Rai 57100, Thailand

[4]Microbe Interaction and Ecology Laboratory, BIOTEC, NSTDA, 113 Thailand Science Park, Pathum Thani, 12120, Thailand

[5]Department of Health Sciences, Faculty of Science, University of Mauritius, Reduit, 80837, Mauritius

*email: tingchiwen@yahoo.com

Supplementary Table S1. Sources of isolates and GenBank accession numbers used in the paper

**Current name**	**Voucher**	**SSU**	**ITS**	**LSU**	**rpb2**	**tef1**	**rpb1**	References
O. agriotidis	ARSEF 5692	DQ522540	JN049819	DQ518754	DQ522418	DQ522322	DQ522368	Kepler et al.[53]
O. barnesii	BCC 28560	EU408776			EU418599		EU408773	Luangsa-Ard et al.[54]
O. barnesii	BCC 28561	EU408775			EU408772		EU408774	Luangsa-Ard et al.[55]
O. barnesii	MFLU 17-1395	MG266438		MG266439	MG271929	MG271932		This study
O. brunneipunctata	OSC 128576	DQ522542		DQ518756	DQ522420	DQ522324	DQ522369	Spatafora et al.[55]
O. capitata	OSC 71233	AY489689			DQ522421		AY489649	Castlebury et al.[37]
O. cf. acicularis	OSC 128580	DQ522543		DQ518757	DQ522423	DQ522326	DQ522371	Spatafora et al.[55]
O. fracta	OSC 110990	DQ522545		DQ518759	DQ522425	DQ522328	DQ522373	Spatafora et al.[55]
O. japonica	OSC 110991	DQ522547	JN049824	DQ518761	DQ522428	DQ522330	DQ522375	Spatafora et al.[55]
O. ophioglossoides	OSC 106405	AY489691			DQ522429		AY489652	Castlebury et al.[37]
O. ravenelii	OSC 110995	DQ522550		DQ518764	DQ522430	DQ522334	DQ522379	Spatafora et al.[55]
O. stylophora	OSC 111000	DQ522552	JN049828	DQ518766	DQ522433	DQ522337	DQ522382	Spatafora et al.[55]
O. variabilis	ARSEF 5365	DQ522555		DQ518769	DQ522437		DQ522386	Spatafora et al.[55]
P. agaricus	YHHPA1305	KP276655	KP276651		KP276667	KP276659	KP276663	Wang et al.[9]
P. agaricus	YHCPA1303	KP276657	KP276653		KP276669	KP276661	KP276665	Wang et al.[9]
P. aurantiacus	MFLUCC 17-2113	MG136904	MG136916	MG136910	MG136870	MG136875	MG136866	This study
P. aurantiacus	MFLUCC 17-2114	MG136905	MG136917	MG136911	MG136871	MG136874		This study
P. aurantiacus	MFLU 17-1394	MG136906	MG136918	MG136912	MG136872	MG136876	MG136867	This study
P. aurantiacus	MFLU 17-1393	MG136907	MG136919	MG136913	MG136873	MG136877	MG136868	This study
P. formosus	ARSEF1424	KF049615	KF049661	KF049634	KF049671	KF049689	KF049651	Kepler et al.[5]
P. kanzashianus			AB027371					Nikoh et al.[56]
P. lianzhouensis	HIMGD20918		EU149921					Zhang et al.[57]
P. lianzhouensis	GIMYY9603	KF226249	EU149922	KF226250		KF226252	KF226251	Zhang et al.[57]
P. marginaliradians	MFLU 17-1582	MG136908	MG136920	MG136914	MG271931	MG136878	MG136869	This study
P. marginaliradians	MFLUCC 17-2276	MG136909	MG136921	MG136915	MG271930	MG136879		This study
P. nipponicus	BCC:1682	KF049620	KF049664	KF049638		KF049694		Kepler et al.[5]
P. nipponicus	NBRC:101406	JN941753	JN943301	JN941388			JN992487	Schoch et al.[58]
P. onorei	BRA: CR23902		KU898841					Crous et al.[10]
P. onorei	BRA: CR23904		KU898843					Crous et al.[10]

**Current name**	**Voucher**	**SSU**	**ITS**	**LSU**	**rpb2**	**tef1**	**rpb1**	References
P. ramosopulvinatus			AB027372					Nikoh et al.[56]
P. ramosopulvinatus	EFCC:5566		KF049658	KF049627		KF049682	KF049645	Kepler et al.[5]
P. ramosus	RCEF6016		KC782530					Crous et al.[10]
P. ramosus	NBRC:109983		AB925946					Crous et al.[10]
P. sinensis	HMAS:43720		NR_119928	NG_042573				Wang et al.[6]
P. tomentosus	BL4	KF049623	KF049666	KF049641	KF049678	KF049697	KF049656	Kepler et al.[5]
P. yunnanensis	YHCPY1005		KF977848		KF977854	KF977850	KF977852	Wang et al.[8]
P. yunnanensis	YHHPY1006		KF977849		KF977855	KF977851	KF977853	Wang et al.[8]
Pu. lilacinum	CBS 284.36	NG_061025	NR_111432					Luangsa-Ard et al.[62]
Pu. lilacinum	CBS 431.87		HQ842812		EF468940	EF468791	EF468897	Luangsa-Ard et al.[63]

Supplementary Table S2. Morphological comparison of asexual morph species of the genus *Polycephalomyces*

Species	Host	Habitat	Synnemata (mm)	Phialides (μm)	Conidia (μm)	Reference
P. aurantiacus	Coleopteran larvae *& O. barnesii*	Buried in the soil	Emerging after 30 days, solitary or not solitary, branched or unbranched, 1.3–2.2 long, showing 1–2 radiating ring-like distributions	α-phialides 10.4–18.3 × 0.8–1.8, narrowly lageniform. β-phialides 22.9–64.2 × 1–1.5, lanceolate or narrowly lageniform	α-conidia 1.8–2.2 × 1.4–1.9 globose to subglobose, yellow slimy in mass. β-conidia 3.2–3.9 × 1.4–1.8 fusiform, one-celled, catenate	This study
P. marginaliradians	On a Cossidae larva	Buried in the soil	Emerging after 14 days, single or branched into 2 or 3 branched, 3.2–4.6 long, showing 1–2 radiating ring-like distributions	α-phialides 11–14.4 × 1.2–1.8, elongated lageniform, caespitose, palisade-like, monoverticillate, branched into 2 phialides, 3 branched on one metula; β-phialides 12.8–23.9 × 1.8–2.7, solitary, growing from hyphae, narrow slender to narrow lageniform, with or without metula at the base	α-conidia 1.9–2.6 μm diam, globose, catenate, one-celled, pale yellow slimy in mass. β-conidia 3.1–3.9 × 1.6–2.1 μm fusiform, one-celled	This study
P. agaricus	Ophiocordyceps sp. *&* melolonthid larvae	Buried in the soil	Solitary, unbranched, agaricshaped, size (0.34– 1.2 × 0.11–0.42); conidial mass pileus-like, light yellow to pale brown, size (0.08–0.25 × 0.36–0.99)	α-phialides lanceolate, length (30.7–81.9), base width (1.3–2.4), neck width (0.5–1.1); β-phialides narrowly lageniform or subulate, length (4.9–28.6), base width (1.8–3), neck width (0.4–0.8)	α-conidia globose to subglobose, size (2–3.1 × 1.8–2.9); β-conidia fusiform, catenate or clump together, size (3.8–6.8 × 1.7–3.2)	Wang et al.[9]

Species	Host	Habitat	Synnemata (mm)	Phialides (μm)	Conidia (μm)	Reference
P. ditmarii	On *Paravespula vulgaris*		2 to 3 distinct branches, 15–25 long, yellowish-white, darkening at the base; Each branch is surmounted by a small subsurface capitulum, 0.5–1 in diameter, with an irregular surface, dotted with numerous small blisters of orange-yellow color, more or less covered with a fine whitish powder	Elongated, cylindrical, attenuating at the top, measuring 20–37 × 1.5–2.5 (3)	Conidia globose to subglobose, smooth, hyaline, measuring 2.2–3 (3.4) × 1.3–1.6	Van Vooren et al.[28]
P. formosus	Coleopteran larvae *& O. barnesii*	On the ground or buried in soil	Solitary, caespitose, branched or unbranched, up to 20 × 0.38 in size, conidial mass light yellow to caramel brown, diam (up to 0.15)	Cylindrical, subulate, length (10–15), base width (1.5–2), neck width (*ca.* 0.5)	α-conidia ovoid, size (2–2.8 × 1.6–2); β-conidia fusiform, catenate, size (3.2–4.8 × 0.8–1.6)	Seifert[16], Bischoff et al.[17]
P. lianzhouensis	On a Lepidoptera larva	In fallen leaves.	Unbranched or dichotomously branched, 10–20 tall, 0.5–1.5 wide, conidial mass not seen	In whorls or intercalary and terminal, terminally awl-shaped, length (6–12), base width (1.3–1.7), neck width (0.5–0. 8)	Ellipsoidal, oblong to cylindrical, size (5–7 × 1.3–1.6)	Wang et al.[7]
P. paludosus	On a Lepidoptera larva		Capitate, 10–20 long 0.5–0.8 thick, cinnamon brown, branched, the branches at right angles, 1–4 long, 0.1–0.2, thick, the branches and the upper poortions of the stems slightly pulverulent	Subulate, 12–20 long, 1–1.5 wide at the base, phialides occurring scattered on the branches below the heads, ventricose, occasionally stellate above, 10.5–14.7 × 1.5–2	Conidia produced singly, hyaline, obovoid, 1.8–2.5 × 1.1–1.3 um covered by a mucus, agglutinating.	Mains[29]

Species	Host	Habitat	Synnemata (mm)	Phialides (μm)	Conidia (μm)	Reference
P. ponerae	Ant (*Ponera Latreille*)	In an ant nest	Simple, 5–10 long, 0.23–0.3 wide, emerged from multiple sites on host, fawn brown at base, white on the top, without obviously inflated ball, secondary synnemata cylindrical	α-phialides awl-formed (7.6–11.9 ×1.1), mostlyon the top of synnemata; β-phialides *Akanthomyces*-like, slightly inflated at base, tapering up into a slenderneck, 5.4–11.9 × 2.2, always on the middle of stipe	α-conidia ellipsoidal (2.2–3.2 × 1.1), aggregate into distinct glutinous spore mass; β-conidia ellipsoidal to cylindrical or fusiform (2.2–4.3 × 1.1), forming dry conidial short chain	Liang et al.[11]
P. ramosus	Lepidopteran larvae *& Hirsutella guignardii*	On the ground or buried in soil, often found in caves	Solitary, crowded or caespitose, unbranched or branched, a size of up to 20 × 1, conidial mass yellow to orange-yellow, diam (0.15–1)	α-phialides cylindrical to narrowly lageniform, length (7–24), base width (1–2), neck width (*ca.* 0.5); β-phialides narrowly lageniform or subulate, length (6–27), base width (2–3.5), neck width (0.5–1)	α-conidia ovoid, size (2.4–3.2 × 1.6–2.4); β-conidia fusiform, catenate, size (3.2–4 × 1.6–2)	Seifert[16], Bischoff et al.[17]
P. sinensis	Lepidopteran larvae *& O. sinensis*	Buried in soil	Solitary, crowded, branched or unbranched, a length of up to 50–60 in culture, conidial mass yellow or yellow-orange	Lanceolate or narrowly lageniform, length (12.5–66), base width (1.4–3.5), neck width (0.6–1.8)	α-conidia ovoid, size (1.7–2.6 × 1.3–2); β-conidia fusiform, catenate or clump together, size (3.3–4.5 × 1.3–2)	Chen et al.[18], Wang et al.[6]
P. tomentosus	On myxomycetes		Fructification a synnema		Conidia globose, 0.7–1 um diam	Seifert[16]

Species	Host	Habitat	Synnemata (mm)	Phialides (μm)	Conidia (μm)	Reference
P. yunnanensis	Hemipteran adults & *O. nutans*	On the ground	Solitary, caespitose or crowded, branched or unbranched, size (0.7–14 × 0.05–1.2); conidial mass white to yellow-brown, size (0.2–1.5 × 0.2–1.4)	α-phialides cylindrical to subulate, length (20.1–57.8), base width (1–2.3), neck width (0.5–1.3); β-phialides narrowly lageniform or subulate, length (7.1–30.6), base width (2.3–3.7), neck width (0.5–1.1)	α-conidia subglobose, ellipsoidal, size (1.4–2.5 × 1.2–2.2); β-conidia fusiform, catenate or clump together, size (2.8–5.7 × 1.1–2.7)	Wang et al.[8]

Supplementary Table S3. Morphological comparison of sexual morph species of the genus *Polycephalomyces*

Species	Host	Habitat	Stromata	Stipe (mm)	Fertile part (mm)	Perithecia (µm)	Asci (µm)	Ascospores (µm)	Part-spores (µm)	Reference
P. marginaliradians	On larvae of Cossidae	Buried in the soil	30–35 long, 2–4.5 mm diam, mostly single, stipitate, unbranched or branched brown to yellow	Cylindrical,1–20 long, 2–3 diam, brown to yellow, with one or two fertile head	4–4.2 long, 0.3–0.45 diam, capitate, lateral, globose to subglobose, pale yellow to yellow, with protruding ostiolar necks	676–803 × 246–328, immersed, yellow, flask-shaped	459–556 × 3.1–4.3, 8-spored, hyaline, filiform, with1.4–2.5 × 2.2–3.2 apical cap	As long as the asci, easily breaking into part-spores, filiform	3.2–4.2 × 1.3–1.7 cylindrical, straight	This study
P. kanzashianus	On Cicadidae		Stipitate, branched, polycephalous	Cylindrical, 30 long, 3–5 diam, leather, rough, terminal branched	Apical or either side spherical, 2–5 in diam., yellow, small	Entirely immersed, flask-shaped, 900–1050 × 270–300	Asci 3 um thick, apical cap 3 um diam	Break into part-spores	3–5 × 1 both truncate	Kobayasi and Shimizu[59]
P. lianzhouensis	On a Lepidoptera larva	In fallen leaves.	Numerous, simple, fleshy, arising from the head, abdomen, and back of the host	Cylindrical, reddish brown or cinnamon colored, 2–12 long, 0.5–1 wide	Hemispherical or capiform, pale yellow or pale yellowish brown, acrogenous, 1–2 wide, 1 high	Narrowly ovoid with protruding apices, vertically immersed, 355–473 × 158–197	Asci: cylindrical, 89–194 × 2–4; caps: hemispherical, 2–3 thick, 2 high	Filiform, break into part-spores	Cylindrical, 3.9–7.8 × 1	Wang et al.[7]

Species	Host	Habitat	Stromata	Stipe (mm)	Fertile part (mm)	Perithecia (µm)	Asci (µm)	Ascospores (µm)	Part-spores (µm)	Reference
P. nipponicus	On Cicadidae	Buried in the soil	Solitary or arranged in twos or threes 20–70 mm in height, often highly branched and polycephalous	Cylindrical simplex, irregularly branched sometimes base has two parts	Terminal or lateral, depressed, fleshy, sometimes crowded, 1–2.5. in diam	Immersed flask-shaped or ovoid, 800–950 × 300–370		Filiform, disarticulating into part-spores	2.8–4.8 × 0.7 truncate	Kobayasi[60]
P. onorei	On caterpillar (*Arctinae*)	Buried in soil, half buried, or among leaves and debris	Numerous, solitary, simple or 2–3 times branched, 10–25 × 0.5–1.5 mm, ampulliform, thickened at the base, cinnamon brown, darker when wet, fading with age and drying to greyish brown	Cylindrical	Subapical, forming lateral pads around stipe, pale brown to ochraceous orange, with sterile apical part	Immersed, 854–950 × 330–395, pyriform, with dark brown protruding apices	Asci 450–510 long	Filiform, cylindrical, breaking to part-spores	(3.5–)4(–5.5) × 0.5–1, truncate, bacilliform	Crous et al.[10]
P. ramosopulvinatus	Nymph of Cicada		Stroma 100 mm long	Cylindrical, leathery, 3.6–4.5 wide, apically branching, glabrous, pallid ocherous in color	Fertile area cushion shaped to globose, with aggregated perithecia forming composite heads	Pyriform, 750–925 × 275–300	3.5–5 wide, with apical cap diameter 3–5	Filiform,disarticulating into partspores	3 × 1 truncate	Kobayasi and Shimizu[59]

Supplementary Table S4. Primers used for current study and optimized PCR protocols

Locus	Primers	Optimised PCR protocols	Approximate size of the PCR amplicons obtained	References
ITS	ITS4: 5’-TCCTCCGCTTATTGATATGC-3’ ITS5: 5’-GGAAGTAAAAGTCGTAACAAGG-3’	(94 ˚C: 30 s, 51 ˚C : 50 s, 72 ˚C: 45 s) × 33 cycles	500 bp	White et al.[34]
SSU	NS1: 5’-GTAGTCATATGCTTGTCTC-3’ NS4: 5’-CTTCCGTCAATTCCTTTAAG-3’	(94 ˚C: 30 s, 51 ˚C : 1 min, 72 ˚C: 2 min) × 33 cycles	1000 bp	Sung et al.[61]
LSU	LROR: 5’-ACCCGCTGAACTTAAGC-3’ LR5: 5’-TCCTGAGGGAAACTTCG-3’	(94 ˚C: 30 s, 51 ˚C : 1 min, 72 ˚C: 2 min) × 33 cycles	800 bp	Sung et al.[61]
EF1-α	EF1-983F: 5’-GCYCCYGGHCAYCGTGAYTTYAT-3’ EF1-2218R: 5’-ATGACACCRACRGCRACRGTYTG-3’	(94 ˚C: 30 s, 58 ˚C : 1 min 20 s, 72 ˚C: 1 min) × 33 cycles	1000 bp	Castlebury et al.[37]
RPB1	CRPB1A: 5’-CAYCCWGGYTTYATCAAGAA-3’ RPB1Cr: 5’-CCNGCDATNTCRTTRTCCATRTA-3’	(94 ˚C: 30 s, 55 ˚C : 30 s, 72 ˚C: 1 min) × 33 cycles	800 bp	Castlebury et al.[37]
RPB2	fRPB2-5f: 5’-GAYGAYMGWGATCAYTTYGG-3’ fRPB2-7cR: 5’-CCCATRGCTTGYTTRCCCAT-3’	(94 ˚C: 30 s, 54 ˚C : 40 s, 72 ˚C: 1 min 20 s) × 33 cycles	1000 bp	Sung et al.[36]
